# Chances and risks in medical risk communication

**DOI:** 10.3205/000211

**Published:** 2015-07-09

**Authors:** Ulrich Hoffrage, Michael Koller

**Affiliations:** 1 Faculty of Business and Economics, University of Lausanne, Lausanne, Switzerland; 2 Centre for Clinical Studies, University Hospital Regensburg, Regensburg, Germany

**Keywords:** risk communication, mammography screening, Bayes’ theorem, probabilities, natural frequencies, relative risk reduction, survival rates

## Abstract

Communication between physicians and patients in everyday life is marked by a number of disruptive factors. Apart from specific interests, mistakes, and misunderstandings on both sides, there are main factors that contribute to the risk in risk communication. Using the example of mammography screening, the current work demonstrates how the meaning of test results and the informative value of measures taken to reduce risk are often misunderstood. Finally, the current work provides examples of successful risk communication.

## Zusammenfassung

Kommunikation, die im Alltag zwischen Ärzten und Patienten stattfindet, wird durch eine Reihe störender Faktoren begleitet. Neben spezifischen Interessen sind es vor allem Fehler und Missverständnisse, die von beiden Seiten zum Risiko der Risikokommunikation beitragen. Am Beispiel des Mammographie-Screenings wird dargestellt, wie die Bedeutung von Testergebnissen und die Aussagekraft von Maßnahmen, die zur Reduktion von Risiken ergriffen werden, oft missverstanden wird. Ferner wird gezeigt, wie eine gelungene Risikokommunikation aussehen kann.

## Patients and physicians sit in the same boat

Everyone knows the advice “*As regards to risks and side effects, ask your doctor or pharmacist*”. Strictly speaking, this sentence does not just constitute a good piece of advice for the readers of drug advertisements but, instead, is used by pharmaceutical companies to protect themselves against possible damage claims. As this demonstrates, risk and side effects are bad, everyone wants to avoid them, and risk communication can have various facets.

On the one hand, people do not want to become patients, and patients do not want to develop any side effects of drugs. On the other hand, physicians and pharmaceutical companies want to avoid making mistakes and being misunderstood or sued. Yet, risk avoidance is not the only common aim. Both sides are interdependent: While patients need the knowledge provided by experts, the different players within the health care system are not only there for the patients but also because of them. In other words, without patients, they would not have anything to do, and their professions would not even exist. Furthermore, each side wants to trust the other side: Distressed patients place their trust in physicians before the treatment, particularly because physicians usually provide the expected help, and physicians may rely on the gratefulness of their cured patients.

Although patients do not always get better after treatment, the commonalities continue even then. Sometimes, physicians make mistakes or do not exclusively act in the best interest of the patient because of constraints or conflicts of interest. Because many patients are aware of this problem, the trust they associate with physicians is sometimes mixed with a pinch of mistrust that is often intensified by the respective communication: regarding risks and side effects when interacting with physicians, patients often ask other patients. But also patients make mistakes, for example, by ignoring instructions or advice and by unfairly holding the physician responsible for the deterioration of their condition, which leads to legal disputes from time to time. For this reason, also physicians have reason to report on risks and side effects when dealing with patients.

## Risks of risk communication

The commonality between physicians and patients, that is, the possibility to help or hurt each other, can also be observed in risk communication. Laypeople are often unable to assess how dangerous their condition is and what could be done about it. Experts are able to help in this respect by providing the relevant information. This is the great chance of risk communication [1]. However, beware of the statement made by Benjamin Franklin “In this world nothing can be said to be certain, except death and taxes”. Even information provided by experts may be incorrect. Sometimes, such information is just incomplete or ambiguous, and it is not always clear if the physician or the patient is to be held responsible for the misunderstanding. Mistakes and possible misunderstandings, such as these, are the risks of risk communication.

Ideally, the knowledge divide between physicians and patients leads to the transparent transfer of relevant information. The situation in daily life, however, is often different because risk communication is not free of disruptive interests [2]. On the one hand, medical care can only be provided when costs are covered. On the other hand, it is legitimate and fully understandable if physicians and caregivers as well as authorities or hospital managements want to protect themselves against legal disputes. Additionally, the manner of risk communication is also influenced by institutional aspects. In our opinion, the perhaps most serious challenge in transparent and understandable risk communication is the inability of some advisors to understand and convey figures correctly. We will present three different examples of how certain values and statistics that are commonly used in risk communication can be misunderstood and show how the risk of such misunderstandings could be reduced.

## The meaning of test results

A 52-year-old woman accepted an invitation to undergo mammography screening. Despite the absence of relevant symptoms, the woman received a suspicious finding – requiring further examinations to clarify if the suspicious lump was indeed breast cancer. In 2011, about 4.9 million women aged between 50 and 69 years received an invitation to undergo mammography screening in Germany. Of the 2.7 million women accepting this invitation, 8.6% of the about 800,000 women who were screened for the first time and 3.2% of the about 1.9 million women for whom this was not their first mammography were asked to present for further examination [3].

However, what is the exact meaning of such a re-invitation? The most frequent reason is a positive finding at the first mammography screening. How high is the probability that it is really breast cancer? This probability, the so-called positive predictive value (PPV), depends on three different parameters. In a survey conducted in the United States, the participating physicians received the following information in this context [4]:

a) *Prevalence: * The probability that a symptom-free woman aged 52 years has breast cancer (B+) is 1%.

b) *Sensitivity: * If a symptom-free woman aged 52 years has breast cancer, the probability that she will receive a positive mammography (M+) result is 80%.

c) *Specificity: * However, if a symptom-free woman aged 52 years does not have breast cancer (B-), the probability that she will still receive a positive mammography result is 10%. 

**Figure 1:**
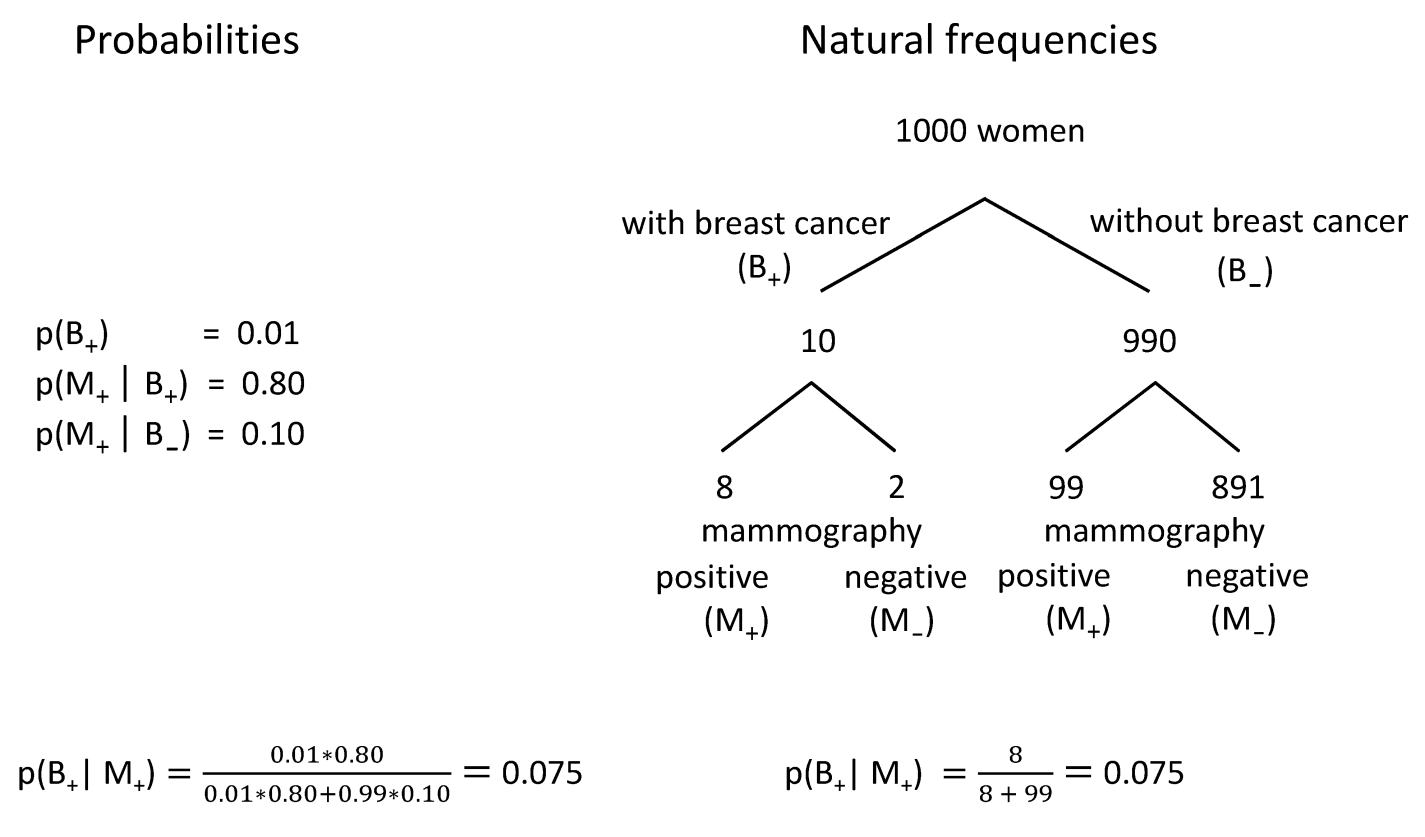
Representation of the same information in terms of probabilities and natural frequencies (adapted from [30]).

Based on this information, 95 of the 100 physicians interviewed concluded that – after a positive screening result – the probability of having breast cancer is between 70% and 80%. However, inserting the three given values into Bayes’ theorem (see left side of Figure 1 (Fig. 1)) reveals that the PPV is 7.5%! The difficulty of calculating the correct PPVs by means of the given information has been shown in several studies that included physicians [5], medical students [6] and laypeople [7] (see [8] for an overview).

In individual cases, misjudgements may lead to serious errors in decision-making regarding further diagnostics and therapy. Such misjudgements may be avoided if natural frequencies instead of probabilities are used to communicate relevant information. Natural frequencies are the number of different cases occurring in a representative random sample [7]. Usually, the conditional probabilities presented within text books have been derived from natural frequencies. Conversely, probabilities can be easily (re-)translated into natural frequencies. In a first step, *prevalence* is related to a fictitious number of people (in the following, the number of 1000 is used) to calculate the number of people affected by the disease in the random sample (1% of 1000 equals 10; Figure 1 (Fig. 1), right hand side). In a second step, the number of affected patients receiving a positive result is determined by means of the *sensitivity* of the test (80% of 10 equals 8). Finally, the number of positive results in the group of healthy people is identified by means of the *specificity* of the test in a third step (the rate of false alarm of 10% in relation to 990 equals 99). Thus, 107 in 1000 women receive a positive result (8+99), but only 8 of these 107 women actually have breast cancer. The quotient 8/107 is 7.5%, and thus the PPV already mentioned above as a result of Bayes’ theorem. Strictly speaking, natural frequencies can also be viewed as applying this rule to a fictitious basic population.

A number of studies [5], [6], [7] (for further examples see [9]) on communicating relevant information have shown that the application of natural frequencies instead of probabilities results in an about threefold increase (from maybe 15% or 20% to approximately 50%) in the percentage of correct conclusions (conclusions consistent with Bayes’ theorem). A teaching unit, in which medical students were instructed on how to translate probabilities into natural frequencies and subsequently how to extract the correct solution from there turned out to be much more effective than the traditional method according to which students were introduced to Bayes’ theorem and instructed how to insert the respective probabilities [10].

Many women accept their invitation to mammography screening because they hope for a negative result and thus for ‘peace of mind’. Are such expectations justified? Figure 1 (Fig. 1) shows that 893 negative results are to be expected in our fictitious random sample. Here, two types of negative results need to be distinguished: First, breast cancer is overlooked in 2 of 10 women affected by this type of cancer (B+), and, secondly, 891 of the 990 healthy women (B-) receive a correct negative result (2+891=893). Thus, for a woman who could be 99% sure to be not affected by breast cancer without undergoing mammography screening (1 – prevalence), this probability increased by 0.78% to 99.78% (=891/893) after the receipt of a negative result. Representation by means of natural frequencies thus helps people understand that even a negative result cannot be equated with security (99.78% does not equal 100%) and that the gain is only marginal (here: 0.78 percentage points).

## Risk reduction

Natural frequencies are not only helpful for interpreting positive test results but also for deciding on the implementation of a certain type of diagnostics or therapy. Should women accept the invitation to mammography screening? Should men have their PSA level determined for early detection of prostate cancer? Should people undergo bypass interventions to reduce the risk of heart failure? What is the benefit in comparison to the risks and disadvantages? The main benefit of such medical interventions is risk reduction, for instance, to die of breast or prostate cancer or to have a heart attack. The question is thus: What are the risks without a diagnostic or therapeutic intervention compared to the risks if the intervention was taken?

Let’s have a look at the figures for mammography screening. Without screening, 4 in 200 healthy women aged between 50 and 69 years who have not been diagnosed with breast cancer will die of this disease within this period of twenty years [11]. If all women would undergo mammography screening, one less woman would die of breast cancer in this period. The most common ways to communicate the reduction in risk are as follows:

a) Relative risk reduction (RRR) amounts to 25%: In 1 of 4 women (=25%), death by breast cancer can be prevented.

b) Absolute risk reduction (ARR) amounts to 0.5%: Only 3 in 200 women instead of 4 in 200 women die from breast cancer; 1 in 200 (=0.5%) women can be saved. 

c) Number-needed-to-screen (NNS) amounts to 200: A total of 200 women have to undergo mammography screening to find the one women benefitting in terms of surviving the next ten years. Not only the effectiveness of screening methods but also that of therapeutic interventions may be evaluated this way. For therapeutic interventions, this measure has been coined number-needed-to-treat (NNT) and is determined as follows: ARR equals 1/NNT (corresponding to 1/NNS for screening methods).

Note the large difference between the communicated values (25%, 0.5%, and 200). Further note that these values are based on the same data, which often leads to considerable confusion. Which of the three values is relevant for a woman who has received an invitation to mammography screening? She is 1 in 200, so that undergoing the screening procedure only reduces her individual risk by 0.5% – which of course, also applies to the other 199 women who have received the same invitation. The figure of 25% exclusively refers to the 4 women who would die of breast cancer without screening – and nobody knows at this point in time who the 4 women are. If they were known, the screening procedure would not be necessary. 

RRR values are commonly used for communicating diagnostic, therapeutic or preventive measures [12], [13]. Whereas ARR values tend to be low as a rule, RRR values are usually high. The use of RRR values in expert literature, the general press and patient information suggests relatively high benefits, but this measure is irrelevant in individual cases. Most people do not understand this value correctly [9], and its application is particularly questionable when the diagnostic, therapeutic, or preventive measures also involve risks [14]. In such cases, the people seeking advice may have decided not for but against the implementation of the measure if risk reduction had been communicated in a more transparent way. A Swiss survey [15] including 53 women showed that most women highly overestimated the benefit of mammography screening and were hardly aware of the risks (false positive results and overtreatment, that means, treatment of patients who have a type of cancer that will be correctly diagnosed but that would never be detected clinically and hence should better not be treated). After the women had been informed in a clear and transparent manner on RRR and ARR as well as on the relation of these two values, the spontaneous readiness to participate in mammography screening in this study dropped from 68% to 11%. 

## The explanatory power of survival rates

Another measure which is often used to quantify the benefit of screening programmes is survival rate, mostly the 5-year survival rate. Related to this, Rudy Giuliani, the former Mayor of New York, hit the headlines in 2007. During his electoral campaign and in the context of his candidacy for the office as the President of the United States, Giuliani compared the benefits of the American health care system with those of the British health care system: “I had prostate cancer five, six years ago. My chance of surviving prostate cancer – and, thank God, I was cured of it – in the United States? 82%. My chance of surviving prostate cancer in England? Only 44% under socialized medicine” [16]. After the comparison of the two figures, Giuliani drew a superficially plausible but nevertheless incorrect conclusion. On closer examination, the difference between the two 5-year survival rates had nothing to do with the nationalisation of the health system but with the fact that a screening programme for prostate cancer was available in the United States but not in Britain.

But the conclusion that the availability of a screening programme would reduce the mortality rate would also be wrong. Screening programmes enable early detection of many cancer diseases, but early diagnosis does by no means imply that death can be postponed.

Let’s take, as a fictitious example, triplets who simultaneously develop clinically apparent prostate cancer at the age of 83 years and die of the disease at the age of 86 years. The first of the triplets does not participate in any screening programme and dies three years after the spontaneous diagnosis of the disease. The second of the triplets undergoes a PSA test at the age of 80 years, followed by a biopsy and the diagnosis of prostate cancer. The third of the triplets has the luck to meet a unique person at the age of 20 years who is able to tell him on the basis of the form of his earlobes that he has prostate cancer. The disease will not break out for another 63 years but he will die from it after 66 years. What is the triplet’s contribution to study results regarding the 5-year survival rate? The first of the triplets will not survive the spontaneous diagnosis by 5 years, and the second is still alive 5 years after the diagnosis was made by means of the PSA test. The case of the third triplet would enhance the reputation of earlobe diagnostics, not only with regard to the 5-year survival rate but also with regard to the 50-year survival rate – a measure unknown in clinical practice. The Lead-time bias affects the three diagnostic methods with their different survival rates differentially: In our fictitious example, such statistics make spontaneous diagnosis look like the worst method and earlobe diagnosis appear to be the best method. But the fact that the respective survival rates do not allow for any statements on mortality is often overlooked. Regardless of when and why the triplets learn about the diagnosis of cancer, each of them dies the same year.

Lead-time bias is further enhanced by overdiagnosis bias. Screening programmes not only bring forward the time of diagnosis but also further the detection of slowly growing types of cancer, which may never metastasise or manifest clinically. 60% to 80% of men are assumed to develop prostate cancer [17]. Most men do not know about their condition and die of other reasons. What would happen with 5-year survival statistics if such comparatively harmless types of cancer could be detected by means of a highly sensitive test? Such overdiagnoses (correct but rather irrelevant and superfluous diagnoses without any life-extending effects) would push up 5-year survival rates and make the method of diagnosis look rather successful, even if the diagnosis does not at all influence the time of death.

Survival statistics are a good measurement tool for comparing effects of cancer therapies in randomised studies. However, such statistics are useless for comparing groups of patients whose disease was diagnosed by different means (early diagnosis vs. symptom-based discovery). “5-year survival rates are artificially inflated by bringing forward the time of diagnosis and by including tumours with a favourable prognosis. In reality, however, this inflation does not necessarily reduce mortality rates. For this reasons, 5-year survival rates are unsuitable for estimating the effect of early diagnoses” ([16], p 4). Most physicians are unaware of these relations. In one of the respective studies, the percentage of physicians who were able to correctly explain lead-time bias and overdiagnosis bias was less than 10% [18]. 

Sound knowledge of these distortions seems to be indispensable for estimating the benefits of screening programmes. For a woman diagnosed with breast cancer by means of early detection mammography, Welch and Frankl calculated a probability rate of 13% that death by breast cancer will be avoided because of early diagnosis ([19]; this calculation is based on an assumed reduction in mortality of 20%). In view of such a low probability rate, the authors concluded that “Most women with screen-detected breast cancer have not had their life saved by screening. They are instead either diagnosed early (with no effect on their mortality) or overdiagnosed.”

## Successful risk communication

The above-mentioned prevalence of breast cancer and test parameters for mammography screening were taken from a U.S. publication of 1982 [4]. We would like to explicitly state that both sensitivity and specificity of mammography screening largely depend on the framework conditions under which programmes are carried out. Significantly less diagnoses of breast cancer will be overlooked and considerably less false positive findings will occur in quality-assured, systematically conducted screening programmes in which analyses are carried out by specially trained and experienced radiologists than in small gynaecological practices.

**Figure 2:**
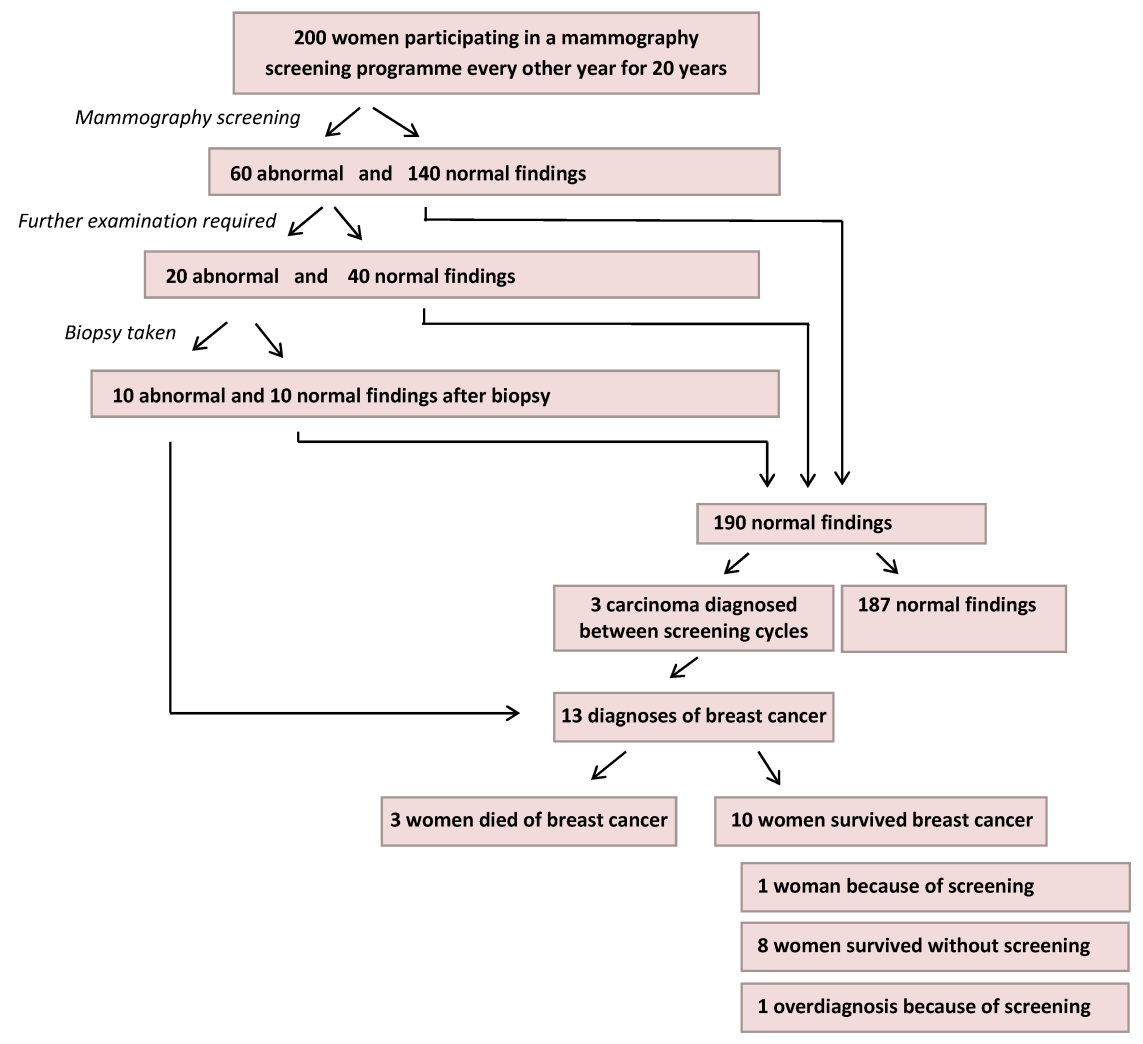
Benefits of mammography screening (adapted from [11])

Improvements can be observed not only with regard to the figures themselves but also with regard to the manner of their communication. We would like to conclude our article by showing such a positive example. Unfortunately, the number of good examples is rather low (the overview of information material on mammography screening presented in [12] and [13] is rather sobering), but some change is on the way. The presentations designed by the Mammography Cooperative (Kooperationsgemeinschaft Mammographie) in collaboration with the German Cancer Research Centre (Deutsches Krebsforschungszentrum) can be viewed as exemplary, and they are adopted by many physicians and journalists. In these presentations, figures are presented as natural frequencies throughout: the diagnostic properties of the screening (PPV and cases of cancer overlooked) and its benefit (risk reduction) and risks (false alarms and overdiagnoses) relate to one and the same fictitious basic population and are thus directly comparable (see Figure 2 (Fig. 2)). We would like to add that the authors of these presentations are very familiar with the results of studies such as the one mentioned above.

The author put the overview shown in Figure 2 (Fig. 2) into words ([11], p. 23), which have been published in an information brochure for the general public ([20], p. 10).

The following figures, which are based on experiences made in other countries and on scientific investigations, shall give you a clear idea of how the benefits and risks are roughly distributed within the entire program:

- Of 200 women participating in a mammography screening programme every other year for 20 years, 140 do not receive a suspicious finding. The remaining 60 women require further examination.
- 40 of these 60 women receive a normal finding when further examined, but the remaining 20 women are advised to have a biopsy taken.
- for 10 of these 20 women the suspicion was not confirmed, and the other 10 women receive the diagnosis breast cancer within the screening programme. Over the 20-year period, 3 of the remaining 190 women also receive the diagnosis of breast cancer but between two screening circles.
- 3 of the overall 13 women with the diagnosis of breast cancer die of the disease, and 10 women do not.
- *1 of these 10 women would not have learned about her diagnosis of breast cancer without the mammography screening programme; 8 women would have been successfully treated, even without participating in the screening programme but some of them would have required a more arduous course of treatment. 1 in 200 women is saved from death by breast cancer because of her regular participation in the mammography screening programme.*

This overview meets all criteria of the catalogue compiled by the specialist team for patient information of the German Network for Evidence-based Medicine (DNEbM), which was developed to support physicians in counselling patients on early cancer diagnosis [21], [22]. The overview also corresponds with the demands for better risk communication in the context of screening programmes [23]. The overview is transparent, and the manner of communication of the most important figures allows for a direct comparison of benefits and risks. This way, every woman is able to decide, either by herself or after consultation with her physician, if she would like to participate in the lottery – also termed mammography screening (further commendable presentations are available in [24], [25], [26]. We would like to encourage physicians, expert societies, patient organisations, health insurances and authorities to take up this example and compile further transparent overviews on the diagnosis and treatment of diseases. The Harding Centre for Risk Literacy at the Max-Planck-Institute for Human Development in Berlin refers to such overviews with the term ‘fact box’ (see also [27] and [28]) and has already produced a number of these boxes [29]. Risks are unavoidable. They have always been and will always be around – but poor risk communication and misunderstanding are really unnecessary. 

## Notes

### Competing interests

The authors declare that they have no competing interests.
